# Development of an Indirect ELISA to Detect Equine Antibodies to *Theileria haneyi*

**DOI:** 10.3390/pathogens10030270

**Published:** 2021-02-27

**Authors:** Reginaldo G. Bastos, Kelly P. Sears, Kelcey D. Dinkel, Lowell Kappmeyer, Massaro W. Ueti, Donald P. Knowles, Lindsay M. Fry

**Affiliations:** 1 Department of Veterinary Microbiology & Pathology, College of Veterinary Medicine, Washington State University, Pullman, WA 99164, USA; reginaldo_bastos@wsu.edu (R.G.B.); kellyp.sears@wsu.edu (K.P.S.); kelcd23@hotmail.com (K.D.D.); massaro.ueti@usda.gov (M.W.U.); dknowles@wsu.edu (D.P.K.); 2 Animal Disease Research Unit, USDA-ARS, Pullman, WA 99164, USA; Lowell.kappmeyer@usda.gov

**Keywords:** equine theileriosis, *Theileria haneyi*, enzyme-linked immunosorbent assay (ELISA), serology

## Abstract

The apicomplexan parasite *Theileria haneyi* is one of two known causative agents of equine theileriosis. It causes milder clinical disease than its more virulent counterpart, *Theileria equi*, in experimentally infected horses, and can superinfect *T. equi*-positive horses. The current equi merozoite antigen 1 (EMA1)-based competitive enzyme-linked immunosorbent assay (ELISA)used in the U.S. to detect equine theileriosis detects *T. equi* but not *T. haneyi*, and the complexity of molecular assays precludes widespread use for epidemiologic studies. In order to facilitate urgently needed studies on the prevalence of *T. haneyi*, the goal of this study was to develop a sensitive and specific serologic assay for the diagnosis of *T. haneyi* based on the equi merozoite antigen 11 (*Th*EMA11). To achieve this objective, *Th*EMA11 was recombinantly expressed in eukaryotic cells and its antigenicity assessed using sera from *T. haneyi*-experimentally infected horses. Confirmation of sera reactivity enabled design and optimization of an indirect ELISA. Specificity of the ELISA for *T. haneyi* was assessed using a cohort of sera from horses experimentally infected and confirmed PCR-positive for either *T. equi* or *T. haneyi*. Data from field samples further demonstrate that the *Th*EMA11 ELISA is capable of identifying *T. haneyi* antibodies in horses from multiple continents around the world.

## 1. Introduction

*Theileria haneyi* is an apicomplexan hemoparasite and one of two known causative agents of equine theileriosis. *T. haneyi* appears to have a global distribution, with infected equids having been identified in North America, South America, and Africa [1,2,3,4,5]. The organism causes milder clinical disease (variable fever, anemia) than *T. equi* in experimentally infected horses, and is capable of superinfection with *T. equi* [3,6]. Horses remain persistently infected following the acute stage of disease, and these asymptomatic horses are presumed to be reservoirs of infectious organisms for competent tick vectors. Unfortunately, while the antiparasitic drug imidocarb diproprionate (ID) resolves the majority of equine infections with U.S. strains of *T. equi*, *T. haneyi* does not appear to be susceptible to ID, and co-infection of horses with *T. equi* and *T. haneyi* reduces the efficacy of ID against *T. equi* [7].

Initial investigation into the serologic immune response to *T. haneyi* revealed that sera from *T. haneyi*-infected horses react with affinity purified, *T. equi* (Florida isolate) equi merozoite antigens (EMA) 1 and 2 [6]. Interestingly, genomic analysis revealed that the *T. haneyi* genome lacks the *ema1*, *3*, and *4* genes, but contains three novel EMA family members, designated *ema11-13* [3]. Antigenic cross-reactivity is attributed to high amino acid identity within the EMA family, both within the *T. equi* genome and between the *T. equi* and *T. haneyi* genomes [3,8]. The EMA family has garnered significant attention in the veterinary diagnostic community, and regulatory *T. equi* serologic assays approved by The World Organization for Animal Health (OIE) and the United States Department of Agriculture (USDA) are based on the equid immune response to the EMAs. The globally validated EMA1-based *T. equi* competitive enzyme-linked immunosorbent assay (ELISA) detects a wide range of global isolates [9,10,11]. However, despite the antigenic cross-reactivity observed via immunoblot, the assay does not detect *T. haneyi* infected horses [3,6]. Currently, PCR-based diagnostic assays are the only available means of definitively confirming *T. haneyi* infection [3,7]. Unfortunately, these molecular diagnostic assays are currently confined to research laboratory use as further optimization is needed to validate the sensitivity of these assays in field-infected animals and to interpret negative results [12].

Due to the aforementioned challenges of molecular assays that currently preclude widespread field use, serology has been the diagnostic assay of choice for detection of infection by the causal agents of equine theileriosis and babesiosis [13]. Therefore, the objective of this study was to develop an indirect ELISA capable of detecting horses infected with *T. haneyi*. To achieve this objective, EMA11, an EMA protein exclusive to *T. haneyi* (*Th*EMA11) was selected. Not only would use of this protein allow discrimination between *T. equi* and *T. haneyi* infection, since the EMA family is specific to equine *Theileria* sp. [8], its use allows differentiation between *T. haneyi* and *Babesia caballi* infection as well. EMA11 was recombinantly expressed and purified, and sera from known *T. haneyi-*infected and uninfected horses were utilized for assessment of specific antibody reactivity with recombinant *Th*EMA11. Confirmation of sera reactivity enabled assay optimization, and indirect ELISA performance was screened against a cohort of horses experimentally infected with either *T. equi* or *T. haneyi* to assess specificity. Data from field samples further demonstrate the *Th*EMA11 indirect ELISA can identify *T. haneyi* antibodies in horses from multiple continents.

## 2. Results

### 2.1. Cloning and Expression of ThEMA11

**Figure 1:**
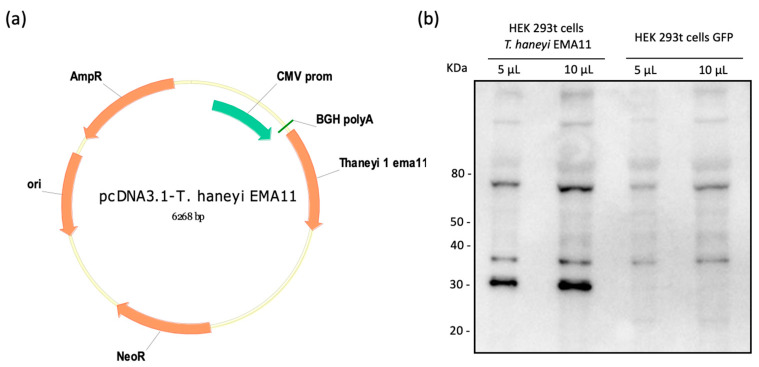
Cloning and expression of *T. haneyi* EMA11 (*Th*EMA11). (**a**) Codon-optimized sequence of *Th*EMA11 was cloned into pcDNA3.1 in frame with the CMV promoter and fused with the 6His tag originating the plasmid pcDNA3.1-*Th*EMA11. (**b**) Expression of recombinant *Th*EMA11 in HEK 293t cells detected by anti-6× His monoclonal antibody.

The nucleotide sequence of *Th*EMA11 was codon-optimized for mammalian cell expression, and successfully cloned into pcDNA3.1 (Figure 1a). The recombinant plasmid (pcDNA3.1-*Th*EMA11) was sequenced to confirm the presence of *Th*EMA11 in-frame with the cytomegalovirus (CMV) promoter (data not shown). Subsequently, HEK 293t cells were transiently transfected with pcDNA3.1-*Th*EMA11, and expression of recombinant *Th*EMA11 was initially confirmed by immunoblot using the anti-6xHis monoclonal antibody (Figure 1b). Results demonstrated the expression of an approximately 33KDa protein, which is the expected molecular weight of *Th*EMA11 fused with the 6× His tag. No protein with similar molecular weight was detected in control cells transfected with pcDNA3.1-GFP (Figure 1b). Additional protein bands with molecular weight ranging from 38 to >80 KDa are also shown in Figure 1b; however, as they are present in both cells transfected with pcDNA3.1 = ThEMA11 or pcDNA3.1-GFP, these are likely non-specific reactions. Once *Th*EMA11 was successfully expressed, we next sought to evaluate the antigenicity of this recombinant *T. haneyi* protein using serologic assays.

### 2.2. Recombinant ThEMA11 Is Recognized by Serum from T haneyi-Infected Horses, but Not Serum from T. equi-Infected or Uninfected Horses

**Figure 2:**
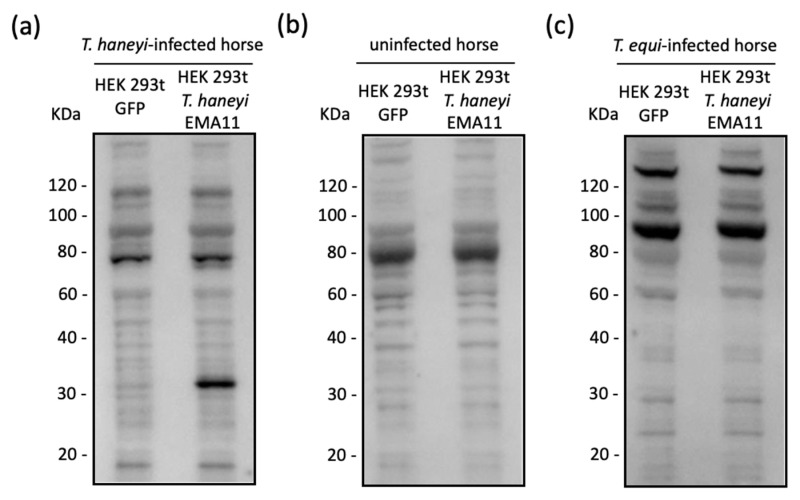
Immunoblot analyses to evaluate the antigenicity of recombinant *Th*EMA11 using sera from horses experimentally infected with *T. haneyi* (**a**), sera from uninfected horses (**b**), and sera from *T. equi* infected horses (**c**). HEK 293t cells expressing GFP were used as a negative control. Blots from one representative animal in each group are shown.

After demonstrating expression of *Th*EMA11 by transiently transfected HEK 293t cells, we next examined whether sera from *T. haneyi*-infected horses recognize the recombinant protein expressed by eukaryotic cells. A crude lysate of the pcDNA3.1-*Th*EMA11-transfected HEK 293t cells was used as antigen for immunoblot analysis. Prior to use in immunoblots, horse sera were adsorbed with HEK 293t-cell lysate to decrease non-specific binding, as described in the Materials and Methods section. Results demonstrated that sera from a horse experimentally infected with *T. haneyi* recognized recombinant *Th*EMA11 (rec *Th*EMA11) (Figure 2a). Sera from an uninfected horses did not detect proteins with the expected molecular weight of *Th*EMA11 (representative uninfected horse serum is shown in Figure 2b). Importantly, sera from *T. equi*- infected horses did not show cross reactivity with *Th*EMA11 (Figure 2c), despite the high level of amino acid identity between the *T. haneyi* protein and members of the EMA family in *T. equi* (Figure S2). The specific recognition of recombinant *Th*EMA11 by *T. haneyi*-infected horse sera, and the absence of recognition of *Th*EMA11 by either uninfected or *T. equi*-infected horse sera prompted us to move forward and use recombinant *Th*EMA11 to develop an indirect ELISA to detect horse antibodies to *T. haneyi*.

### 2.3. Optimization of ThEMA11 as an Antigen for Use in the Indirect ELISA Fformat

**Figure 3:**
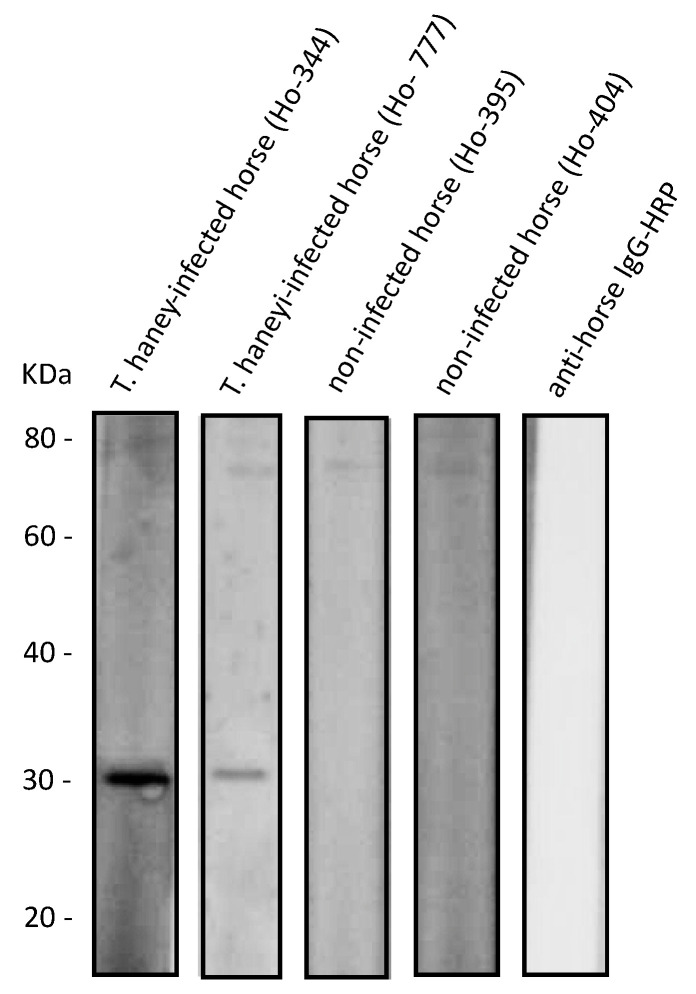
Antigenicity of purified recombinant *Th*EMA11 using sera from *T. haneyi*-infected horses (Ho-344 and Ho-777). Sera from uninfected horses (Ho-395 and Ho-404) were used as negative controls. Rabbit anti-horse IgG-HRP alone was also used as a negative control.

As a first step for ELISA optimization, we scaled up the transient transfection of HEK 293t cells with pcDNA3.1-*Th*EMA11 and purified the recombinant *Th*EMA11 using cobalt resin. The level of purity of recombinant *Th*EMA11 was assessed following each step of the purification process by immunoblot using a monoclonal antibody to the anti-6xHis tag (data not shown). A concentration of approximately 1 μg/μL of rec *Th*EMA11 was obtained after purification. Next, the antigenicity of purified *Th*EMA11 was evaluated using sera from *T. haneyi*-infected horses (Figure 3). The results of this analysis demonstrate that purified, recombinant *Th*EMA11 retained its antigenicity and was specifically recognized by sera from *T. haneyi*-infected horses. Immunoblots using sera from two representative, *T. haneyi* experimentally infected horses, Ho-344 and Ho-777, are shown in Figure 3. Sera from uninfected horses failed to react with purified, recombinant *Th*EMA11. Immunoblots using sera from two representative uninfected animals, Ho-305 and Ho-404, are presented in Figure 3. Similarly, no reactivity was observed when anti-horse IgG HRP secondary antibodies were used alone (Figure 3). 

**Figure 4:**
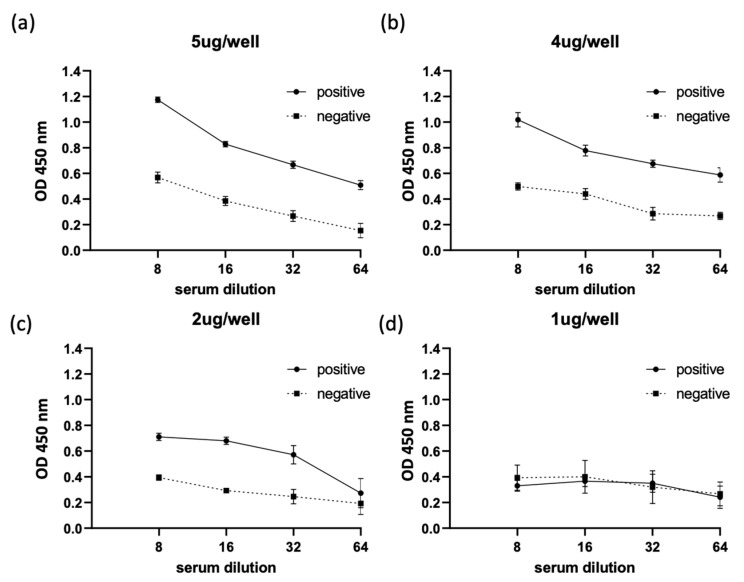
Titration of the optimal amount of recombinant *Th*EMA11 in an ELISA format in relation to horse serum dilutions. Results show titration of serum from one representative *T. haneyi*-infected horse and serum from one representative uninfected horse. Four serial dilutions of the sera (1:8, 1:16, 1:32, and 1:64), and four different *Th*EMA11 recombinant protein concentrations (5 μL/well, 4 μL/well, 2 μL/well, and 1 μL/well) were tested (**a–d**). Error bars represent the standard deviation of technical replicates.

Subsequently, the optimal amount of recombinant *Th*EMA11 was determined in an ELISA format in relation to horse serum dilutions (Figure 4). For this analysis, we used serum from one *T. haneyi*-infected horse and serum from one uninfected horse, selected based on reactivity of the serum with recombinant ThEMA11 in the immunoblots, as described in the previous paragraph. Four serial dilutions of the sera (1:8, 1:16, 1:32, and 1:64), and four different *Th*EMA11 recombinant protein concentrations (5 μL/well, 4 μL/well, 2 μL/well, and 1 μL/well) were tested (Figure 4a–d). Data showed that the best discrimination between positive and negative sera was obtained using 4 μL/well of antigen with a serum dilution of 1:8 (Figure 4b). Therefore, these parameters were used for the *Th*EMA11-based ELISA. OD450nm values below 0.2 were obtained when rabbit anti-horse IgG HRP was used alone (data not shown). In addition, we used the *Rhipicephalus microplus* tick antigen Bm86 fused with the 6-His tag (Bm86-6-His) and purified by cobalt column, a similar approach used to purify *Th*EMA11, to evaluate potential reactivity of horse antibodies to the 6-His tag. No significant reactivity was detected in representative *T. haneyi*-infected and uninfected horse sera against Bm86-6-His, indicating that that 6-His tag is not implicated in the OD signal observed in the *Th*EMA11 ELISA (data not shown).

**Figure 5:**
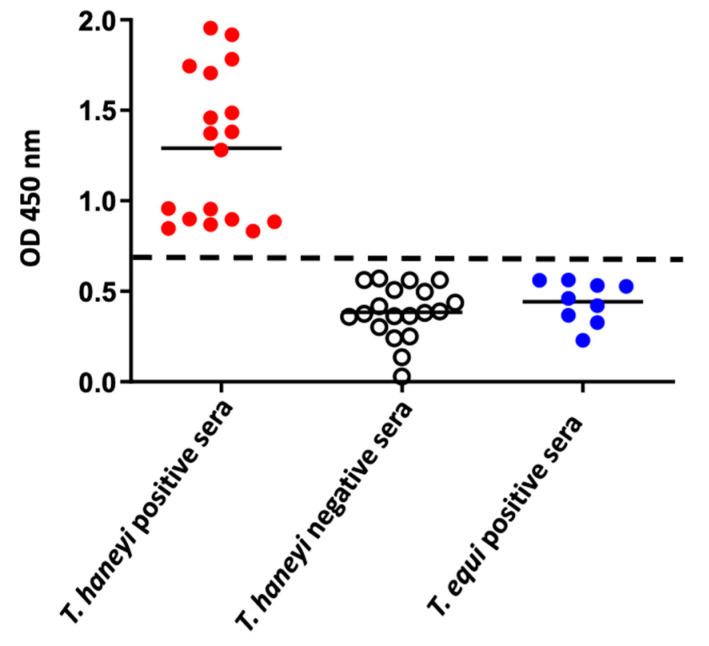
Results of the *Th*EMA11-based ELISA using serum samples from *T. haneyi* experimentally infected horses (n = 18), from *T. equi* experimentally infected horses (n = 9), and from uninfected horses (n = 19). The dashed line indicates the cutoff of 0.8 OD450 nm, representing the average of negative samples (uninfected horse sera) plus three standard deviations. The solid lines represent the average OD for each group.

A total of 18 serum samples from *T. haneyi* experimentally infected horses and 19 serum samples from uninfected horses were then used to determine the *Th*EMA11-based ELISA cutoff (Figure 5). An optimal cutoff of 0.8 OD450 nm was determined, representing the average of negative samples plus three standard deviations. In addition, sera from nine experimentally *T. equi*-infected, known *T. haneyi*-negative horses were also analyzed. As demonstrated, sera from all *T. equi*-infected horses present OD values below the cutoff threshold and therefore, were correctly shown as negative for exposure to *T. haneyi* (Figure 5). Assessment of 35 field-collected horse serum samples (11 known *T. haneyi*-infected and 24 known uninfected horses) demonstrated that the recombinant *Th*EMA11-based ELISA correlates well with immunoblot results and showed relative sensitivity and specificity of the new ELISA of 90.90% and 95.83%, respectively.

### 2.4. Use of the ThEMA11-Based ELISA to Detect Antibodies to T. haneyi in Horse Sera from Different Geographic Regions 

**Figure 6:**
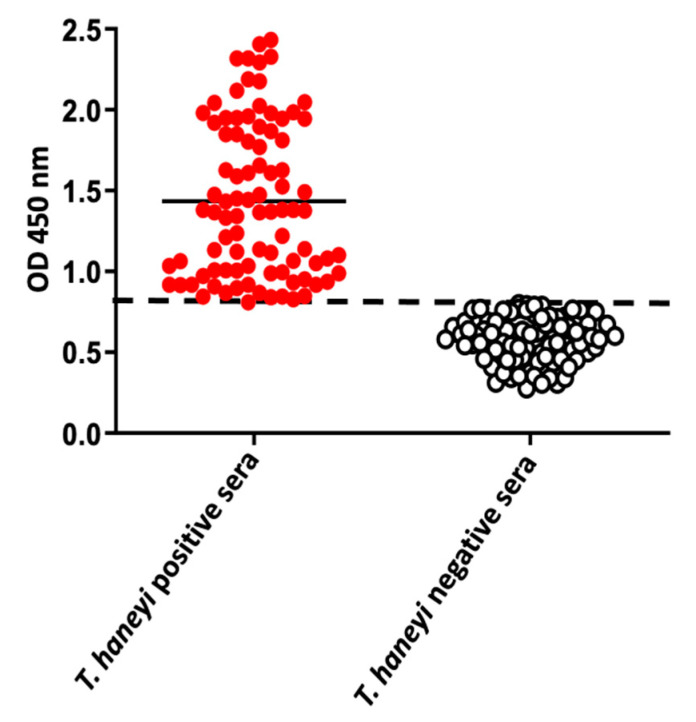
Use of the *Th*EMA11-based ELISA to investigate the presence of antibodies against *T. haneyi* in serum samples (n = 176) from horses residing in or with a travel history to distinct geographical regions endemic for equine theileriosis. The dashed line indicates the cutoff of 0.8 OD450 nm, previously determined representing the average of negative samples (uninfected horse sera) plus three standard deviations. The solid lines represent the average OD for each group. 100/176 equine field serum samples were positive for *T. haneyi*, and 76/176 were negative.

After determining the positive threshold, sensitivity, and specificity of the *Th*EMA11-based ELISA, we used the assay to investigate the presence of antibodies against *T. haneyi* in serum samples from horses residing in or with a travel history to distinct geographical regions (Figure 6). A total of 176 horse sera were tested (145 from U.S., 6 from Germany, 13 from Mexico, 2 from France, 3 from Ireland, 1 from Puerto Rico, 3 from the Netherlands, and three sera from unknown locations). Of the tested samples, 50.56% (n = 90) were positive for *T. haneyi* using the *Th*EMA11 ELISA, and 48.86% (n = 86) were considered negative (Figure 6). These equine serum samples were also assessed for *T. equi* and *B. caballi* antibodies (Table 1 and Table 2). Results demonstrated that 58.62% (51 serum samples) were positive for both *T. haneyi* EMA11 and *T. equi* EMA1. In contrast, 62.96% (51 serum samples) were negative for *Th*EMA11 but positive for the presence of antibodies to *T. equi* EMA1. A total of 27.92% (7 serum samples) were positive both for *Th*EMA11 and *B. caballi* RAP-1 antibodies. On the contrary, 29.41% (5 serum samples) were positive for the presence of *B. caballi* RAP-1 antibodies, but negative for *Th*EMA11. Considering the detection of *Th*EMA11 antibodies in samples that were negative for *T. equi* EMA1 or *B. caballi* RAP-1, together the results also reinforce the specificity of the *Th*EMA11-based ELISA described in this study. Collectively, the results demonstrate the use of the *Th*EMA11 ELISA to investigate the presence of antibodies to *T. haneyi* in horse sera from distinct geographical regions around the world. 

## 3. Discussion

Currently, the only available diagnostic assay for *T. haneyi* is nested PCR, which hinders widespread assessment of prevalence of this recently discovered organism. Despite the reported high sensitivity of PCR, especially when utilizing nested PCR, multiple factors can lead to inhibition of reactions, leading to false negative results [14]. Another downside of direct molecular diagnostic assays, such as PCR, is the potential for false-negative results due to low parasitemia in peripheral blood, especially in the chronic phase of *B. caballi* infections. In addition, molecular assays are relatively more expensive. Therefore, serology remains the more widely accepted diagnostic, especially for persistent infections such as those caused by *T. equi* and *T. haneyi*. 

Since *T. equi* and *T. haneyi* are closely related, careful analysis was required to identify a candidate antigen for use in a *T. haneyi*-specific diagnostic assay. In a previous study, antigenic cross-reactivity was observed to a subset of *T. equi* proteins in immunoblots, but the *T. equi* EMA1 based regulatory diagnostic competitive ELISA failed to detect *T. haneyi* infected horses. For development of a *T. haneyi*-specific serologic assay, the *T. haneyi* EMA11 protein was selected because it is exclusive to *T. haneyi*. The antigen was then carefully evaluated to ensure cross-reactivity did not occur between *T. equi* and *T. haneyi* infected horses as there is a high level of amino acid identity between the *T. equi* and *T. haneyi* EMA family members. Additionally, a mammalian cell expression system was utilized to ensure proper protein folding, and to reduce bacterial protein contamination and resultant high background reactivity. Following expression and purification, the antigenicity of recombinant *Th*EMA11 was verified via screening with sera from known infected and non-infected horses. The infection status of these horses was correlated at multiple timepoints with nPCR and/or serial blood smears, and immunoblot using serum from horses in both the acute and chronic stages of infection. 

Since serum from horses experimentally infected with only one geographic isolate of *T. haneyi* was used for the initial assay development and optimization steps, we chose to screen a cohort of geographically diverse equine serum samples to gain a broader perspective of the potential use of this assay on a global scale. Performance of the assay using this diverse sample set was strong, and high agreement was noted with the *T. haneyi* immunoblot. Using this information, the serologic assay was determined to have a sensitivity and specificity of 90.9% and 95.83%, respectively. It was beyond the scope of this study to investigate the level of conservation of *Th*EMA11 in parasites around the world. However, the fact that the *Th*EMA11-based ELISA was able to detect antibodies in geographically diverse equine serum samples suggest that EMA11 is highly immunogenic and conserved among worldwide isolates of *T. haneyi*.

A reliable serologic diagnostic assay for *T. haneyi* will become crucial for international movement of horses as recently published data suggests that co-infection with *T. haneyi* reduces the efficacy of ID against *T. equi* [7]. Furthermore, ID lacks efficacy in horses infected with *T. haneyi* alone [7]. This data is concerning, as potential new anti-*Theileria* chemotherapeutic compounds are currently only in early testing phase, leaving a paucity of potential therapeutic options for infected horses [15,16,17]. Additionally, although experimental infection with *T. haneyi* appears to be mild, the clinical signs and severity associated with natural infection remains unknown, and could be more significant, as has been documented with *T. equi.*

The assay developed in this study will enable subsequent, global evaluation of the prevalence of *T. haneyi.* Following more extensive validation, use of this assay in screening horses prior to inter-country movement may help with the continued control on equine theileriosis on a global scale. 

## 4. Materials and Methods

### 4.1. T. haneyi EMA11 Cloning, Expression, and Purification

*T. haneyi* parasites and the *Th*EMA11 sequence used in this study were previously described in [3,8]. The full-length sequence of *Th*EMA11 was codon-optimized for mammalian cells, and a 6His tag added to its C-terminal end (GeneArt, Thermo Fisher Scientific, Waltham, MA, USA) (Figure S1). The synthetic gene was then cloned into pcDNA3.1 and the plasmid, termed pcDNA3.1-*Th*EMA11, was then used to express the target protein in human embryonic kidney (HEK) 293t cells (ATCC^®^, Gaithersburg, MD, USA).

For the expression of recombinant *Th*EMA11, HEK 293t cells were transiently transfected with pcDNA3.1-*Th*EMA11 using polyethylenimine (PEI) transfection reagent per standard protocols. Briefly, HEK 293t cells were seeded overnight in six-well plates (70 to 80% confluence) and transfected with a target plasmid using PEI (1µg plasmid DNA: 4 µL PEI ratio), as previously described [18]. Four hours after transfection, the transfection mix was replaced with complete Dulbecco’s modified essential medium (cDMEM) (10% fetal bovine serum, 24 mM of HEPES, 2 mM of L-glutamine, 100 IU/mL penicillin, and 100 µg/mL streptomycin). 48 h after transfection, cells were collected in 1× Cell Culture Lysis Reagent (Promega, Madison, WI, USA) containing the Halt Protease Inhibitor Single Use Cocktail, EDTA-Free (Thermo Fisher Scientific). Lysate of transfected HEK 293t cells were stored at −80 °C until use for immunoblot analysis and protein purification.

Recombinant *Th*EMA11 was purified using the HisPur™ Cobalt Purification Kit following the manufacturer’s protocol (Thermo Fisher Scientific). Briefly, transient transfection of HEK 293t was scaled up to be performed in 150 cm^2^ cell culture flasks. Forty-eight hours post-transfection, the culture supernatant was removed, and cells re-suspended in 1× PBS containing Halt Protease Inhibitor Single Use Cocktail, EDTA-Free. Cells were then lysed in three freeze-thaw cycles and stored at −80 °C until purification. For protein purification, cobalt columns were equilibrated to room temperature and washed in Equilibration/Wash Buffer provided by the HisPur™ purification kit. The lysate of pcDNA3.1-*Th*EMA11-transfected HEK 293t cells was loaded into the columns and incubated for 30 min at 4 °C on a rocking platform. Columns were then centrifuged at 700× *g* for 2 min, and the resin washed twice in Equilibration/Wash Buffer. Target protein was eluted by adding one resin-bed volume of Elution Buffer provided by the HisPur™ purification kit followed by centrifugation at 700× *g* for 2 min. Elution steps were repeated twice, and fractions were stored at −80 °C until use. 

### 4.2. Horse Serum Samples

Serum samples from *T. haneyi* experimentally infected horses (n = 13) [3,6,7] were collected at multiple time points following infection and used as positive controls. Serum samples (n = 19) from healthy horses located in WA or ID, USA (where *T. equi* and *T. haneyi* are not naturally present in equine populations), which were confirmed negative for *T. haneyi* via nPCR and blood smear cytology were used as negative controls in this study. The known *T. haneyi*-positive and negative sera were used to determine the *Th*EMA11-based ELISA cutoff. In addition, serum samples from confirmed *T. equi*-infected horses (n = 8) were also used to assess the specificity of the *T. haneyi* EMA11-based ELISA. All serum samples were obtained from horses housed at the USDA-ADRU, University of Idaho, or Washington State University animal facilities. All animal experiments were approved by the Washington State University and University of Idaho Institutional Animal Care and Use Committees, ASAF numbers 4973 and 6241 (Washington State University, Pullman, WA, USA) and 2016-18 and 2016-28 (University of Idaho, Moscow, ID, USA).

After establishing the basic positive/negative cutoff and ensuring that *T. equi* infected horse serum did not recognize EMA11, as described above, field horse serum samples (n = 176) from different regions around the world were used to begin testing the assay in a diverse sample set. The serum samples were from horses in the U.S., Mexico, and Germany, among other countries. Serum samples were obtained through collaboration with OIE Reference Laboratory for equine piroplasmosis located at Washington State University. Reactivity of these samples for *T. equi* (168/176) and/or *B. caballi* (43/176)*,* depending on the individual case circumstances, was determined by the Reference Laboratory via commercially available cELISAs and immunoblots as described below. Due to individual horse circumstances, not all samples were assayed for both pathogens (some were tested for just *T. equi,* some for just *B. caballi*, and some for both pathogens). The date of collection and sample source location are presented in Table S1. 

### 4.3. Competitive ELISA (cELISA) for T. equi or B. caballi

Horse sera tested in this study for the presence of anti-*T. haneyi* antibodies were evaluated by cELISA to detect antibodies to *T. equi* and/or *B. caballi* as previously described in Knowles et al 1992 and Kappmeyer et al 1999, respectively [19,20]. 

### 4.4. Immunoblot Analysis

Immunoblots were performed using lysates of HEK 293t transfected with pcDNA3.1-*Th*EMA11 or with purified recombinant *Th*EMA11 as antigen and sera from known *T. haneyi*-infected horses or monoclonal antibody (mAb) recognizing the 6His tag (clone AD1.1.10; Bio-Rad, Hercules, CA, USA). In addition, sera from healthy, uninfected horses and confirmed *T. equi*-infected horses were used as controls in the immunoblots. Briefly, 5 or 10 μL of cell lysate or 5 μL of recombinant purified *Th*EMA11 was loaded into each lane and separated in NuPAGE™ 4–12% Bis-Tris gel (Invitrogen, Waltham, MA USA). The gel was then electrotransferred to a nitrocellulose membrane and blocked with PBS 0.02% Tween 20 (PBS-T) 10% non-fat milk. After blocking, the membrane was incubated for 1 h at room temperature with either horse serum (1:50 or 1:100) or the anti-6His tag mAb (1:500). For immunoblots using lysate of HEK 293t cells transfected with pcDNA3.1-*Th*EMA11, horse sera were adsorbed as follows. Serum samples were incubated for 48 h at 4 °C with lysate of wildtype HEK 293 cells (approximately 10^6^ cells). After that, the mix was centrifuged (1000× *g*, 5 min) and supernatant used for the immunoblots. After washing in PBS-T, the membrane was incubated for 1 h at room temperature with secondary anti-mouse IgG HRP (SeraCare; 1:2500) or anti-horse IgG HRP (SeraCare; 1:2500). The immune complexes were revealed using an enhanced chemiluminescence method (ECL™; Amersham, Buckinghamshire, UK).

### 4.5. Indirect ELISA

Immunlon™ 2 HB 96-well microtiter ELISA plates (Thermo Scientific, Hanover Park, IL, USA) were coated overnight at 4 °C with 50 μL of recombinant purified *Th*EMA11 (4 μg/well) in 1× Coating Buffer (BioLegend, San Diego, CA, USA). After that, excess antigen was removed, and the plates were blocked with 200 μL/well of Blocker™ Casein in PBS (Thermo Fisher Scientific) at room temperature (RT) for 1 h. Following the blocking step, serial dilutions (1:2 to 1:256) of *T. haneyi*-positive or negative horse sera were added to the plates and incubated at RT for 1 h. After five washes in 0.05% (*v*/*v*) Tween-20 in PBS (PBS-T), rabbit anti-horse IgG-HRP (1:10,000) (Millipore-Sigma, St. Louis, MO, USA) was added to each well, and the plates were incubated at RT for 1 h. Plates were then washed five times in PBS-T and developed with 100 μL of 1-Step™ Ultra TMB-ELISA Substrate Solution (Thermo Fisher Scientifics). The enzymatic reaction was stopped via addition of 100 μL of TMB Stop Solution (0.2 M H_2_SO_4_) (SeraCare, Gaithersburg, MD, USA) to each well, and plates read at 450 nm using an ELISA plate reader (MultiSkan MCC, Thermo Fisher Scientific). Immunoblot was used as gold standard to evaluate sensitivity and specificity of the *Th*EMA11-based ELISA. Sensitivity and specificity were calculated using the following formula: sensitivity = [number of true positive samples − number of false negative samples) × 100]/number of true positive samples; and specificity = [number of true negative samples − number of false positive samples) × 100]/number of true negative samples.

## Figures and Tables

**Table 1:** Presence of *T. haneyi* EMA11 and *T. equi* EMA1 antibodies in horse sera from distinct geographic regions.

*T. haneyi* Positive Samples (n = 87)		*T. haneyi* Negative Samples (n = 81)	
*T. equi* positive	*T. equi* negative	*T. equi* positive	*T. equi* negative
58.62% (n = 51)	41.37% (n = 36)	62.96% (n = 51)	37.07% (n = 30)

**Table 2:** Presence of *T. haneyi* EMA11 and *B. caballi* RAP-1 antibodies in horse sera from distinct geographic regions.

*T. haneyi* Positive Samples (n = 87)		*T. haneyi* Negative Samples (n = 81)	
*B. caballi* positive	*B. caballi* negative	*B. caballi* positive	*B. caballi* negative
26.92% (n = 7)	73.07% (n = 19)	24.41% (n = 5)	70.58% (n = 12)

## Data Availability

Data is contained within the article and supplementary material. Additional raw data is available on request from the corresponding author.

## Supplementary Materials

The following are available online at https://www.mdpi.com/2076-0817/10/3/270/s1, Figure S1: Codon-optimized *Theileira haneyi* EMA11 for mammalian expression. Figure S2: Amino acid alignment of *Theileira haneyi* EMA11 and *T. equi* EMA1. Table S1: List of field horse samples.
