# Supplementary material for: Development of an Indirect ELISA to Detect Equine Antibodies to Theileria haneyi
Source: Pathogens. 2021 Feb 27;10(3):270. doi: 10.3390/pathogens10030270 (PMC7997436; doi:10.3390/pathogens10030270)
Supplement: Supplementary file 1 [file pathogens-10-00270-s001.pdf]

Table S1. Internal sample ID, date of collection and location of field horse samples used in this study.

Internal sample ID	Date of collection	Location
#1	2/14/13	USA
#2	2/27/13	USA
#4	4/22/13	USA
#5	4/23/13	USA
#7	5/1/13	USA
#8	5/1/13	Germany
#9	5/1/13	Germany
#10	5/8/13	USA
#11	5/4/13	USA
#12	5/8/13	USA
#13	5/21/13	USA
#14	6/24/13	USA
#15	6/7/13	USA
#16	6/13/13	USA
#17	6/16/13	USA
#18	6/26/13	USA
#19	7/20/13	USA
#21	8/7/13	USA
#22	8/26/13	USA
#23	8/29/13	USA
#24	8/28/13	USA
#30	8/22/13	USA
#31	9/9/13	USA
#32	9/17/13	USA
#33	9/17/13	USA
#34	unknown	USA
#35	9/25/13	Germany
#36	9/25/13	Germany
#37	10/15/13	USA
#39	10/23/13	USA
#40	10/29/13	USA
#41	10/31/13	USA
#42	11/11/13	USA
#43	11/11/13	USA
#45	12/4/13	USA
#46	unknown	Germany
#49	12/16/13	USA

#52	1/21/14	USA
#53	2/3/14	USA
#59	4/18/14	Germany
#62	5/27/14	USA
#63	6/5/14	USA
#64	6/4/14	unknown
#65	7/8/14	USA
#68	8/18/14	USA
#69	9/9/14	USA
#70	9/9/14	USA
#72	9/9/14	USA
#73	9/9/14	USA
#74	9/24/14	USA
#75	10/10/14	USA
#76	10/21/14	USA
#77	10/23/14	USA
#78	10/23/14	USA
#79	10/23/14	USA
#80	10/23/14	USA
#81	10/23/14	USA
#82	11/17/14	USA
#83	11/17/14	USA
#84	11/17/14	USA
#86	12/2/14	USA
#87	12/2/14	USA
#88	12/2/14	USA
#89	12/2/14	USA
#90	12/2/14	USA
#91	1/5/15	USA
#92	1/5/15	USA
#93	1/5/15	USA
#94	1/5/15	USA
#98	1/15/15	Puerto Rico
#100	2/16/15	USA
#101	2/16/15	USA
#102	2/16/15	USA
#103	2/26/15	USA
#106	4/21/15	USA
#107	4/22/15	USA
#108	4/22/15	USA

#109	4/22/15	USA
#110	4/29/15	Mexico
#111	4/29/15	Mexico
#112	4/29/15	Mexico
#113	4/29/15	Mexico
#114	4/29/15	Mexico
#115	4/29/15	Mexico
#116	4/29/15	Mexico
#117	4/29/15	Mexico
#118	4/29/15	Mexico
#119	5/10/15	Mexico
#120	5/18/15	USA
#121	6/1/15	USA
#122	6/10/15	USA
#123	6/10/15	USA
#124	6/10/15	USA
#125	6/10/15	USA
#126	7/29/15	USA
#127	7/29/15	USA
#128	7/29/15	USA
#129	7/29/15	USA
#130	7/31/15	USA
#131	8/13/15	USA
#132	9/16/15	USA
#135	11/17/15	USA
#137	1/4/16	Mexico
#138	12/10/15	USA
#139	3/3/16	USA
#140	3/3/16	USA
#141	3/3/16	USA
#142	3/3/16	USA
#144	5/10/16	USA
#146	1/26/16	USA
#147	unknown	USA
#148	unknown	USA
#149	unknown	USA
#151	3/9/16	USA
#152	3/14/16	USA
#153	unknown	USA
#154	6/13/16	USA

#155	7/13/16	USA
#160	unknown	Germany
#161	8/19/16	USA
#162	9/14/16	France
#164	9/29/16	USA
#166	9/23/16	USA
#167	9/19/16	Ireland
#168	10/6/16	USA
#169	9/28/16	USA
#170	10/20/16	USA
#171	10/20/16	USA
#172	10/20/16	USA
#173	11/8/16	USA
#175	11/21/16	Ireland
#176	12/9/16	USA
#177	12/14/16	USA
#178	12/20/16	USA
#179	1/5/17	USA
#180	1/9/17	USA
#181	1/13/17	USA
#183	4/23/17	USA
#185	3/30/17	USA
#186	4/13/17	USA
#187-1	5/12/17	USA
#188	7/11/17	USA
#189	7/25/17	USA
#190	8/10/17	USA
#191	8/14/17	Mexico
#192	8/11/17	USA
#193	9/11/17	USA
#194	9/20/17	USA
#195	9/20/17	USA
#196	9/21/17	Mexico
#197	10/10/17	USA
#198	10/24/17	USA
#199	11/13/17	USA
#200	11/24/17	USA
#201	11/24/17	USA
#202	11/29/17	USA
#203	12/18/17	unknown

#204	12/18/17	USA
#205	12/18/17	USA
#206	12/18/17	unknown
#207	12/28/17	USA
#208	12/28/17	USA
#209	12/28/17	USA
#210	1/24/18	USA
#211	4/9/18	The Netherlands
#212	10/31/18	USA
#213	11/26/18	USA
#215	1/29/19	USA
#216	unknown	The Netherlands
#217	3/1/19	Ireland
#218	3/20/19	The Netherlands
#219	3/26/19	USA
#220	6/25/19	USA
#221	7/19/19	USA
#222	9/27/19	France
#223	10/30/19	USA

Fig 1S

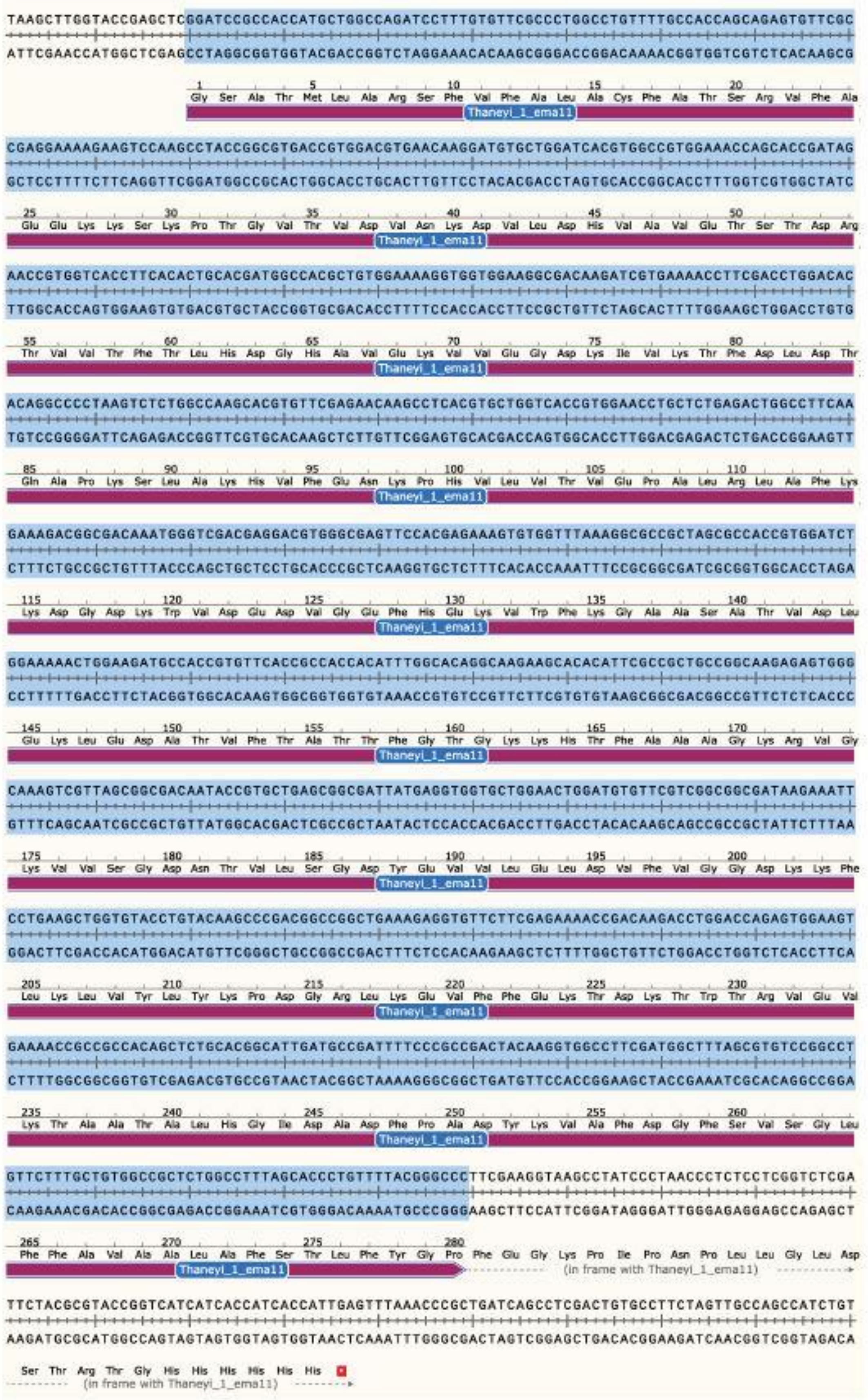

Fig S2

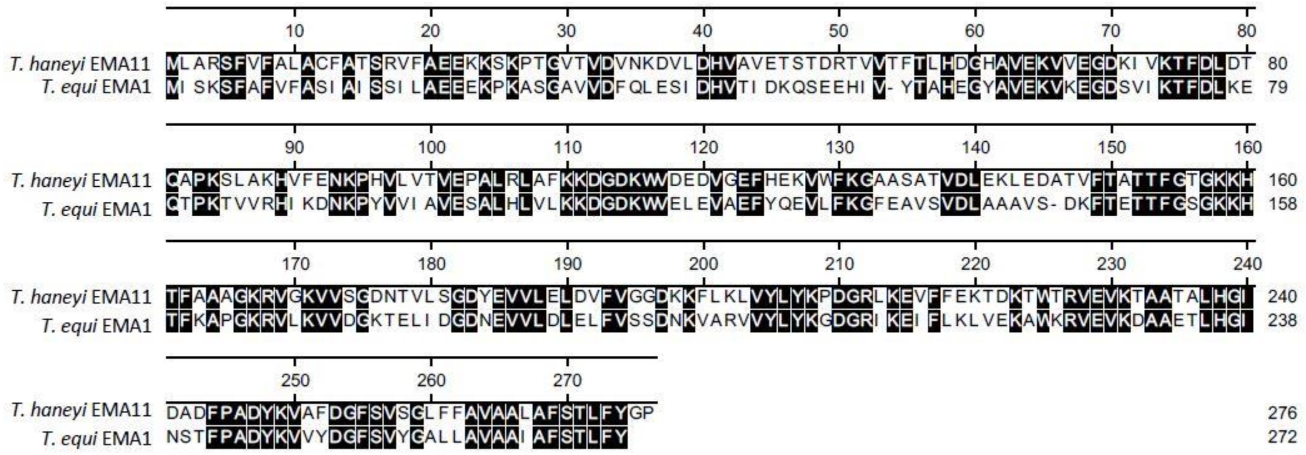
T. haneyi EMA11 MLARSFVFALACFATSRVFAEEKKSKPTGVTVDVNKDVLDHVAVETSTDRTVVTFTLHDGHAVEKVVEGDKIVKTFDLDT 80
T. equi EMA1 MISKSFAFVFASIAISSILAEEEKPKASCAVVDFQLESIDHVTIDKQSEEHIV-YTAHEGYAVEKVKEGDSVIKTFDLKE 79
T. haneyi EMA11 CAPKSLAKHVFENKPHVLVTVEPALRLAFKKDGDKWDEDVGEFHEKVWFKGAASATVDLEKLEDATVFTATTFGTGKKH 160
T. equi EMA1 CTPKTVVRHIKDNKPYVVIAVESALHLVLKKDGDKWELEVAEFYQEVLFKGFEAVSVDLAAAVS-DKFTETTFGSGKKH 158
T. haneyi EMA11 TFAAAGKRVGKVVSGDNTVLSGDYEVVLELDVFVGGDKKFLKLVYLYKPDGRLKEVFFEKTDKTWTRVEVKTAATALHGI 240
T. equi EMA1 TFKAPGKRVLKVVDCKTELIDGDNEVVLDLELFVSSDNKVARVVYLYKGDGRIKEIFLKLVEKAWKRVEVKDAAETLHGI 238
T. haneyi EMA11 DADFPADYKVAFDGFSVSGLFFAVAALAFSTLFYGP 276
T. equi EMA1 NSTFPADYKVVYDGFSVYCALLAVAAIAFSTLFY 272
